# Waardenburg Syndrome Type-II in Twin Siblings: An Unusual Audio-Pigmentary Disorder

**DOI:** 10.7759/cureus.10889

**Published:** 2020-10-10

**Authors:** Sadia Masood, Palwasha Jalil, Naila Ahmed Jan, Muhammad Sadique

**Affiliations:** 1 Dermatology, Aga Khan University Hospital, Karachi, PAK; 2 Dermatology, Bolan Medical College, Quetta, PAK; 3 Nephrology, Aga Khan University Hospital, Karachi, PAK

**Keywords:** waardenburg syndrome, sensorineural deafness, white forelock

## Abstract

Waardenburg syndrome (WS) is an interesting inherited audio-pigmentary disorder. The syndrome shows no gender, racial, or ethnic predilection. This unique disorder is characterized by pigmentary abnormalities, deafness, and neural crest-derived tissue defect. WS can be recognized by some specific clinical features that appear after birth; not all affected individuals possess all the clinical features. It has four clinical sub types based on the mutant gene and characteristic morphology. These morphological features are broad nasal root, white forelock, the difference in the colour of eyes, congenital leukoderma, and sensorineural deafness. We report an interesting case of WS in twin boys who fulfill the criteria of WS-II. Our cases have four major criteria (white forelock, heterochromia, sensorineural hearing loss, first degree relative with WS), and 1 minor criterion to establish the diagnosis of WS-II. Most clinical features of WS-II except sensorineural deafness are benign and do not need any intervention but severe deafness can be a serious problem. The current report is unique and is a rare case of WS in twin infants. We present this case for its rarity, relative paucity of literature, and also to emphasize the clinical presentation of this extremely rare disease in twins.

## Introduction

Waardenburg syndrome (WS) is an unusual audio-pigmentary genetic disorder [1]. The syndrome is named WS after a Dutch ophthalmologist P. J. Waardenburg, who described a rare syndrome comprised of six unique clinical features: broad nasal root, hypertrichosis of medial side of the eyebrows, lateral displacement of the medial canthus, white forelock, partial or total heterochromia iridis, and sensorineural deafness [2]. WS shows no gender, racial, or ethnic predilection. There are four clinical variants and WS-I and WS-II are the most common types [3]. Type I is clinically manifested as congenital deafness (sensorineural), dystopia canthorum (lateral displacement of medial eye corners), neural tube defects, cleft palate and lip with patchy depigmentation of hair and skin [1-2]. These symptoms are associated with pigmentary abnormalities of the eyes. In Type II, the inner canthi of both eyes are normal but have some other features similar to type I. Type-III WS is an extreme presentation of type I with the abnormality of upper limbs, it is a very rare clinical form of WS [4]. Type IV WS in addition to symptoms of type I have features of Hirschsprung disease [2]. As it is a genetic disease, there is no definitive treatment for WS, but supportive treatment with cochlear implants and surgery in case of association with Hirschsprung syndrome can be done [5]. Genetic counseling is necessary. We report here a unique case of WS in twin infants, for its rarity, relative paucity of reported literature, and also to emphasize the clinical presentation of this extremely rare disease in twins.

## Case presentation

**Figure 1:**
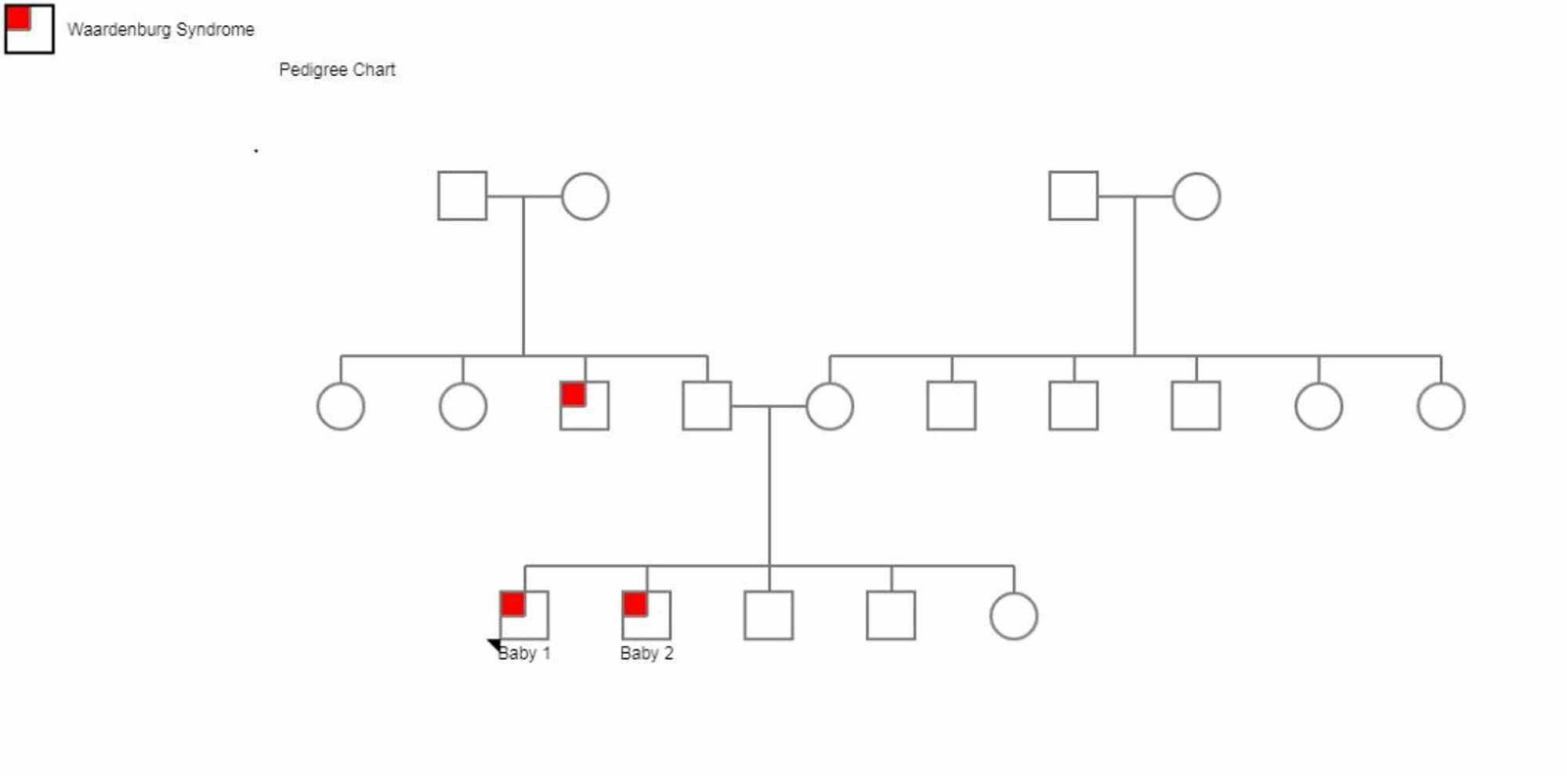
Pedigree chart of the family showing affected paternal uncle and twin siblings

Two twin infant boys were referred from the otolaryngology outpatient clinic (ENT) for the complaint of white patches of hair and pigmentary skin changes since birth. These symptoms were also associated with sensorineural deafness. They were born of consanguineous parents through normal vaginal delivery. Family history revealed the presence of similar skin symptoms and deafness in their paternal uncle (Figure 1).

**Figure 2:**
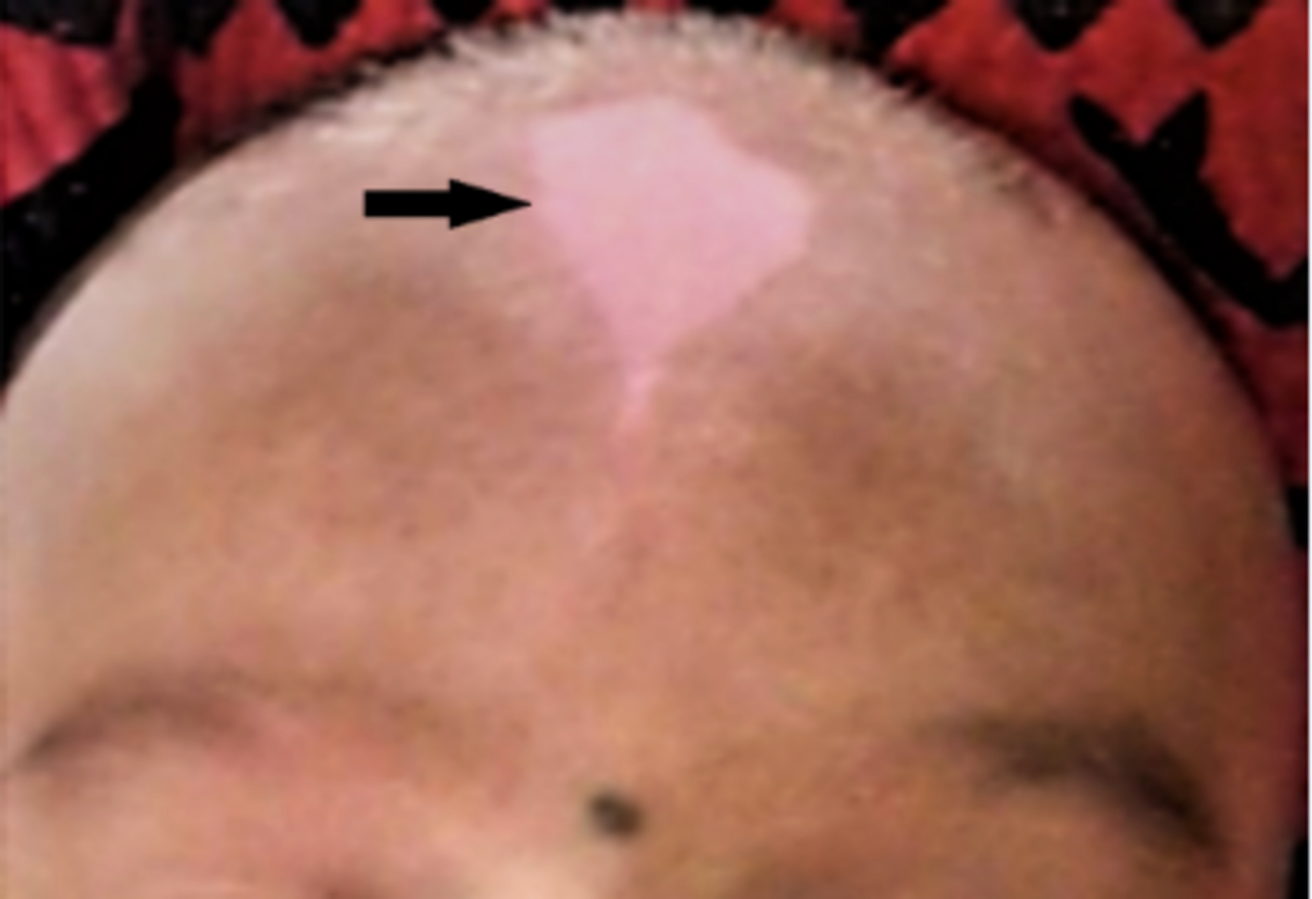
Baby 1 with white patch on the forehead (white forelock)

**Figure 3:**
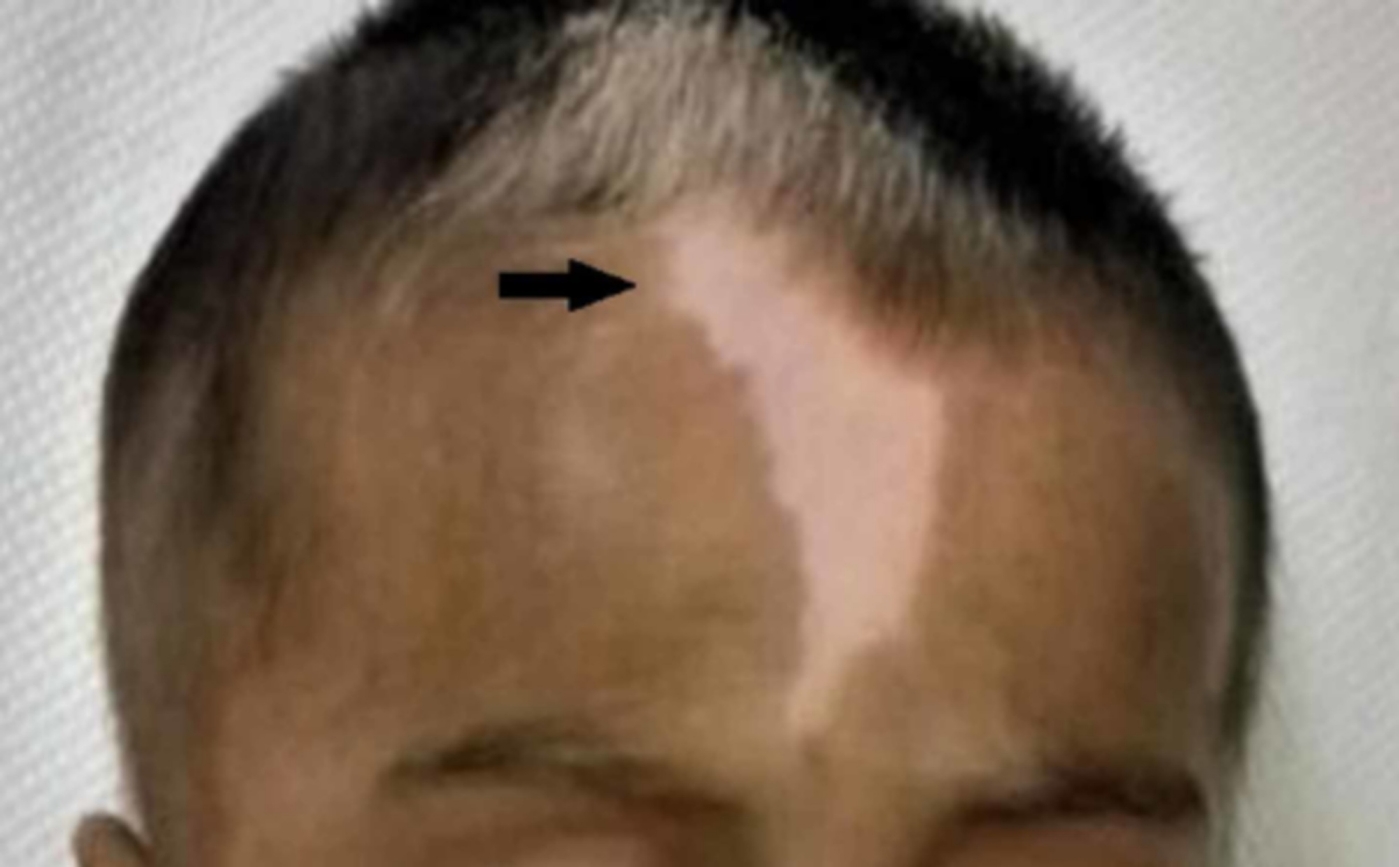
Baby 2 with white patch on the forehead (white forelock)

**Figure 4:**
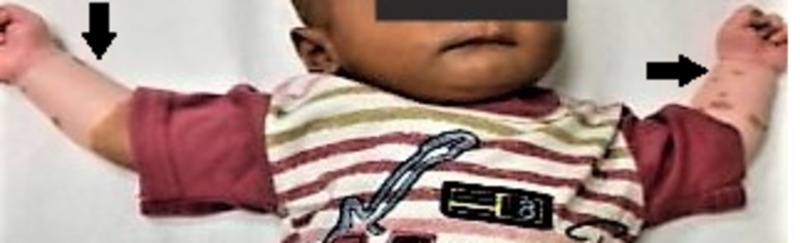
Baby 2 with white leukoderma on upper limbs

They have three elder siblings and all of them are normal. Physical examination revealed white forelock and hypo-pigmented patches on limbs with full heterochromia of iris (not visible as babies were not opening their eyes properly) (Figures 2-4).

Wood's lamp examination revealed complete depigmentation over the affected area. Audiograms of both infants showed severe sensorineural deafness.

Based on history, clinical examinations, and audiometry, they were diagnosed as a case of WS. They had been categorized in type II WS because they have a positive family history, sensorineural deafness, white forelock, and different coloured irises. Systemic examination and routine laboratory workup were normal in both infants. Topical emollients were given. The parents were counseled about the prognosis and association of syndrome with the complications of consanguineous marriages because for a child to have the disease, only one affected gene is necessary to pass on. We advised the parents to regularly follow the ENT outpatient clinic for further management.

## Discussion

WS is a rare disease of great importance, particularly in the pediatric population [6]. This unique inherited disorder is characterized by pigmentary abnormalities, deafness, and neural crest-derived tissue defect [7]. Several different gene mutations (insertion, deletion, frameshifts, missense, and nonsense mutations) can cause WS [8]. During embryogenesis, there is an abnormal distribution of melanocytes, which results in patchy areas of depigmentation [8]. WS is mostly due to the changes in genes in Type 1 and Type 3 due to the point mutations and can be detected by the use of multiplex ligation-dependent probe amplification in specific genes [9]. 

WS can be recognized by some specific clinical features that appear after birth; not all affected individuals possess all the clinical features [2]. It has four clinical subtypes based on the mutant gene and characteristic morphology [10]. These morphological features are broad nasal root, white forelock, the difference in the colour of eyes, congenital leukoderma, and sensorineural deafness [10]. It is a clinical diagnosis, and Waardenburg consortium proposed diagnostic criteria. The major criteria are heterochromia of iris, sensorineural deafness, white forelock, lateral displacement of eyes canthi, and presence of WS in a first-degree relative [1]. The minor diagnostic criteria are broad nasal root, white macules/patches on the skin, synophrys, premature greying of the scalp, and hypoplasia of nasal alae [1]. Two major or one minor and two minor criteria are needed to diagnose WS type I [2]. WS-II is characterized by sensorineural deafness and heterochromia of irides [11]. For diagnosing WS-II, Liu et al. have proposed two major criteria be fulfilled while premature greying replaced the dystopia canthorum [11]. WS-III is similar to WS-I with the addition of few musculoskeletal abnormalities like upper limb contractures and hypoplastic muscles [9]. WS type IV has a strong association with Hirschsprung disease [12]. 

We report an interesting case of WS in twin boys who fulfill the criteria of WS-II. Our cases had four major criteria (white forelock, heterochromia, sensorineural hearing loss, first degree relative with WS), and one minor criterion to establish the diagnosis of WS-II. Most clinical features of WS-II except sensorineural deafness are benign in nature and do not need active intervention but severe deafness can be a serious problem [10]. An early diagnosis with social and vocational training and rehabilitation depending upon the extent of involvement remains the only rescue for these patients [5]. Areas of hypopigmentation may diminish in size or even disappear with time [2]. Most of the clinical types of WS have a good prognosis [1]. Morbidity is related to the defect of neural crest-derived tissues, including mental disability, deafness and ocular disorders (cataracts), and skeletal anomalies [5]. We report a rare inherited disorder that had never been reported in twins. Through this unique case report, we recommend that early diagnosis and prenatal genetic identification is very essential. Spina bifida is a rare manifestation of WS but the severity can make it a relatively important element of the disease [13]. Prenatal screening is advisable through transvaginal ultrasound and to follow the guidelines for high-risk pregnancies to monitor neural tube defects [13].

## Conclusions

We report an unusual and interesting rare genetic disorder. The treatment approach should be multidisciplinary. Genetic counseling is necessary because one affected gene can pass the syndrome to the next generation. As there is no definitive treatment, the family and the patient's awareness regarding symptomatic treatment is also very essential. In such rare cases, early diagnosis and prenatal genetic identification are very essential.
